# A mathematical model for the adenylosuccinate synthetase reaction involved in purine biosynthesis

**DOI:** 10.1186/1742-4682-4-11

**Published:** 2007-02-27

**Authors:** Evgeniya A Oshchepkova-Nedosekina, Vitalii A Likhoshvai

**Affiliations:** 1 Institute of Cytology and Genetics SB RAS, Novosibirsk, Russia

## Abstract

**Background:**

Development of the mathematical models that adequately describe biochemical reactions and molecular-genetic mechanisms is one of the most important tasks in modern bioinformatics. Because the enzyme adenylosuccinate synthetase (AdSS) has long been extensively studied, a wealth of kinetic data has been accumulated.

**Results:**

We describe a mathematical model for the reaction catalyzed by AdSS. The model's parameters were fitted to experimental data obtained from published literature. The advantage of our model is that it includes relationships between the reaction rate, the concentrations of three substrates (GTP, IMP and ASP), the effects of five inhibitors (GMP, GDP, AMP, ASUC and SUCC), and the influence of Mg^2+ ^ions.

**Conclusion:**

Our model describes the reaction catalyzed by AdSS as a fully random process. The model structure implies that each of the inhibitors included in it is only competitive to one of the substrates. The model was tested for adequacy using experimental data published elsewhere. The values obtained for the parameters are as follows: *V*_*max *_= 1.35·10^-3 ^mM/min, *Km*_*GTP *_= 0.023 mM, *Km*_*IMP *_= 0.02 mM, *Km*_*ASP *_= 0.3 mM, *Ki*_*GMP *_= 0.024 mM, *Ki*_*GDP *_= 8·10^-3 ^mM, *Ki*_*AMP *_= 0.01 mM, *Ki*_*ASUC *_= 7.5·10^-3 ^mM, *Ki*_*SUCC *_= 8 mM, *Km*_*Mg *_= 0.08 mM.

## Background

Biosynthesis of the purines AMP and GMP in *Escherichia coli *is a many-staged process supported by a complex network of enzymes. Some of the genes that encode these enzymes are arranged into operons (*purF, purHD, purMN, purEK, guaBA, purB*), while others are located in single cistrons (*purT, purl, purC, purA, guaC*). Expression of these operons is regulated by regulatory proteins (PurR, DnaA, CRP) and various low-molecular-weight compounds [1-3]. The activities of the encoded enzymes are additionally regulated by substrates, reaction products, and certain other low-molecular-weight substances [4,5].

The enzyme adenylosuccinate synthetase (AdSS; GDP-forming IMP: L-aspartate ligase, EC 6.3.4.4), which is the product of the *purA *gene, catalyzes the conversion of IMP to ASUC in the presence of Mg^2+^:

*IMP *+ *GTP *+ *ASP *→ *GDP *+ *PI *+ *ASUC*.

There are many nucleotides that inhibit AdSS. For example, AMP is a competitive inhibitor of IMP; ASUC, of IMP; dGMP, of IMP; GMP, of GTP. GDP is a competitive inhibitor of GTP, which in part explains a gradual decrease in the rate of ASUC formation in solutions if the GTP concentration is not reduced. dAMP, CMP, and UMP can also produce inhibitory effects, albeit much less pronounced [6].

Mathematical models of the reaction catalyzed by AdSS have been proposed in a variety of studies. In 1969, Rudolph and Fromm proposed an equation that includes one inhibitor [7]. It was demonstrated that each of SUCC, GDP, and IMP is a competitive inhibitor of only one substrate and that the molecular mechanism of the reaction catalyzed by AdSS is a rapid equilibrium, fully random process. To describe the dependence of the reaction rate on whether the inhibitor competes against the substrate for binding to the enzyme, an 11-parameter model was proposed. Although the kinetics of the AdSS-catalyzed reaction in the presence of the inhibitors SUCC, GDP, IMP, and ASUC was well studied experimentally, the formula included too many constants and the model constants (including the inhibition constants) were not evaluated.

In 1979, Stayton and Fromm proposed a slightly different equation for one inhibitor [8]. In this case, the inhibition of AdSS by ppGpp was considered. It was demonstrated that ppGpp is a competitive inhibitor of GTP, but not of IMP or ASP. This model also describes the effect of the inhibitor using four inhibition constants, so only the apparent values of these constants were calculated. Interestingly, varying the concentrations of IMP or GTP (at fixed concentrations of the other two substrates) affected the calculated values of the respective inhibition constants.

In 1995, Kang and Fromm investigated the influence of Mg^2+ ^ions on the AdSS-catalyzed reaction [9]. It was demonstrated that for AdSS to be in the activated form, two Mg^2+ ^ions are required. One interacts with the β- and γ-phosphoryl groups of GTP, the other with the aspartate in the enzyme's active center, improving the affinity of the enzyme for ASP. Kinetic experiments on the interactions of Mg^2+ ^and ASP were performed with saturating concentrations of GTP and IMP, so the GTP and IMP concentrations were not included in the model. Although the authors themselves proved that AdSS has two binding centers for Mg^2+^, the model treats the Mg^2+ ^concentration as if there were only one (at least this is how we interpret the presence of ion concentration as an item raised to the first power). The initial velocity in the Hill plot (Fig. 1 in [9]) was measured at saturating concentrations of IMP, GTP and Asp with Mg^2+ ^varying.

**Figure 1:**
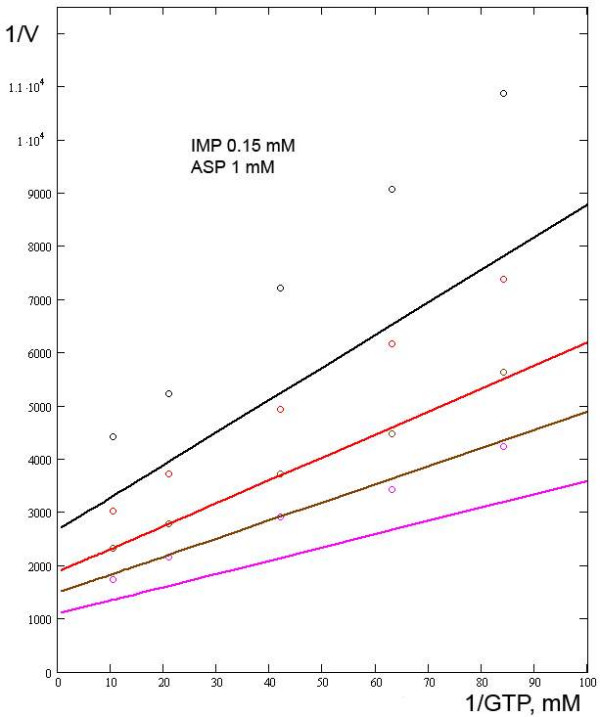
**Relationships between the reaction rate and the concentration of GTP in the presence of SUCC**. SUCC concentrations were (black line and circles) 50 mM; (red line and circles) 25 mM; (brown line and circles) 12.5 mM; (crimson line and circles) 0. Experimental data from [7].

Thus, although a model has been proposed for each of a variety of effectors, there is still no single model that exploits the pool of available kinetic data in its entirety. We report a more complete model, which describes the reaction catalyzed by adenylosuccinate synthetase and includes the concentrations of three substrates (GTP, IMP, and ASP), the effects of five inhibitors (GMP, GDP, AMP, ASUC, and SUCC), and the influence of Mg^2+ ^ions.

## Results

The enzyme AdSS is inhibited by GMP, GDP, AMP, ASUC and SUCC. Enzyme activity requires the presence of Mg^2+ ^ions. Knowing how these effectors work, the reaction rate can be written in a generalized form as follows:

V=Vmax⁡⋅GTPKmGTP⋅IMPKmIMP⋅ASPKmASP(1+GTPKmGTP+GMPKiGMP+GDPKiGDP)⋅(1+IMPKmIMP+AMPKiAMP+ASUCKiASUC)⋅(1+ASPKmASP+SUCCKiSUCC)⋅Mg2+KmMg1+Mg2+KmMg     (1),
 MathType@MTEF@5@5@+=feaafiart1ev1aaatCvAUfKttLearuWrP9MDH5MBPbIqV92AaeXatLxBI9gBaebbnrfifHhDYfgasaacH8akY=wiFfYdH8Gipec8Eeeu0xXdbba9frFj0=OqFfea0dXdd9vqai=hGuQ8kuc9pgc9s8qqaq=dirpe0xb9q8qiLsFr0=vr0=vr0dc8meaabaqaciaacaGaaeqabaqabeGadaaakeaacqWGwbGvcqGH9aqpcqWGwbGvdaWgaaWcbaGagiyBa0MaeiyyaeMaeiiEaGhabeaakiabgwSixpaalaaabaWaaSaaaeaacqWGhbWrcqWGubavcqWGqbauaeaacqWGlbWscqWGTbqBdaWgaaWcbaGaem4raCKaemivaqLaemiuaafabeaaaaGccqGHflY1daWcaaqaaiabdMeajjabd2eanjabdcfaqbqaaiabdUealjabd2gaTnaaBaaaleaacqWGjbqscqWGnbqtcqWGqbauaeqaaaaakiabgwSixpaalaaabaGaemyqaeKaem4uamLaemiuaafabaGaem4saSKaemyBa02aaSbaaSqaaiabdgeabjabdofatjabdcfaqbqabaaaaaGcbaWaaeWaaeaacqaIXaqmcqGHRaWkdaWcaaqaaiabdEeahjabdsfaujabdcfaqbqaaiabdUealjabd2gaTnaaBaaaleaacqWGhbWrcqWGubavcqWGqbauaeqaaaaakiabgUcaRmaalaaabaGaem4raCKaemyta0KaemiuaafabaGaem4saSKaemyAaK2aaSbaaSqaaiabdEeahjabd2eanjabdcfaqbqabaaaaOGaey4kaSYaaSaaaeaacqWGhbWrcqWGebarcqWGqbauaeaacqWGlbWscqWGPbqAdaWgaaWcbaGaem4raCKaemiraqKaemiuaafabeaaaaaakiaawIcacaGLPaaacqGHflY1daqadaqaaiabigdaXiabgUcaRmaalaaabaGaemysaKKaemyta0KaemiuaafabaGaem4saSKaemyBa02aaSbaaSqaaiabdMeajjabd2eanjabdcfaqbqabaaaaOGaey4kaSYaaSaaaeaacqWGbbqqcqWGnbqtcqWGqbauaeaacqWGlbWscqWGPbqAdaWgaaWcbaGaemyqaeKaemyta0KaemiuaafabeaaaaGccqGHRaWkdaWcaaqaaiabdgeabjabdofatjabdwfavjabdoeadbqaaiabdUealjabdMgaPnaaBaaaleaacqWGbbqqcqWGtbWucqWGvbqvcqWGdbWqaeqaaaaaaOGaayjkaiaawMcaaiabgwSixpaabmaabaGaeGymaeJaey4kaSYaaSaaaeaacqWGbbqqcqWGtbWucqWGqbauaeaacqWGlbWscqWGTbqBdaWgaaWcbaGaemyqaeKaem4uamLaemiuaafabeaaaaGccqGHRaWkdaWcaaqaaiabdofatjabdwfavjabdoeadjabdoeadbqaaiabdUealjabdMgaPnaaBaaaleaacqWGtbWucqWGvbqvcqWGdbWqcqWGdbWqaeqaaaaaaOGaayjkaiaawMcaaaaacqGHflY1daWcaaqaamaalaaabaGaemyta0Kaem4zaC2aaWbaaSqabeaacqaIYaGmcqGHRaWkaaaakeaacqWGlbWscqWGTbqBdaWgaaWcbaGaemyta0Kaem4zaCgabeaaaaaakeaacqaIXaqmcqGHRaWkdaWcaaqaaiabd2eanjabdEgaNnaaCaaaleqabaGaeGOmaiJaey4kaScaaaGcbaGaem4saSKaemyBa02aaSbaaSqaaiabd2eanjabdEgaNbqabaaaaaaakiaaxMaacaWLjaWaaeWaaeaacqaIXaqmaiaawIcacaGLPaaacqGGSaalaaa@D836@

where *V*_*max *_is the maximum reaction rate; *GTP, IMP*, and *ASP *are the concentrations of the corresponding substrates; *GMP, GDP, AMP, ASUC*, and *SUCC *are the concentrations of the corresponding inhibitors; *Mg*^2+ ^is the concentration of Mg^2+ ^ions; *Km*_*GTP*_, *Km*_*IMP*_, *Km*_*ASP *_are the Michaelis-Menten constants for the corresponding substrates; *Km*_*Mg *_is the Michaelis-Menten constant for Mg^2+ ^ions; *Ki*_*GMP*_, *Ki*_*GDP*_, *Ki*_*AMP*_, *Ki*_*ASUC*_, and *Ki*_*SUCC*_, are the constants of the efficiency of reaction inhibition by the corresponding substances.

The model's parameters were verified against 61 curves from published data [6,7,9]. Different publications use different values of the rate constant of AdSS: 15600 s^-1 ^[10], 1.47 s^-1 ^[9], 1.0 s^-1 ^[11]. However, since most publications do not indicate the enzyme concentrations used, we calculated the value for *V*_*max *_using our model.

We evaluated the reaction constants in the absence of effectors using experimental results from the work by Rudolph and Fromm [7] and observed good agreement (calculations not shown). The parameter values inferred from the curves were as follows: *V*_*max *_= 1.35·10^-3 ^mM min^-1^, *Km*_*GTP *_= 0.023 mM, *Km*_*IMP *_= 0.02 mM, *Km*_*ASP *_= 0.3 mM.

Rudolph and Fromm, who examined the effect of SUCC in detail [7], proposed that SUCC is competitive to ASP. Our calculations indicate that this assumption is consistent with the kinetic data. The model output and experimental data on how SUCC affects the reaction rate at different concentrations of GTP are presented in Fig. 1. As can be seen from this figure, there is an inconsistency between the model output and experimental data. A possible explanation will be discussed below. Also, we estimated the effect of SUCC on the reaction rate at different concentrations of IMP and ASP and observed good agreement with experimental data (calculations not shown). Using our model, the value of the constant *Ki*_*SUCC *_is 8 mM.

GDP is a competitive inhibitor of GTP. Based on experimental data from the work by Rudolph and Fromm [7], we evaluated *Ki*_*GDP *_as 8·10^-3 ^mM (calculations not shown).

**Figure 2:**
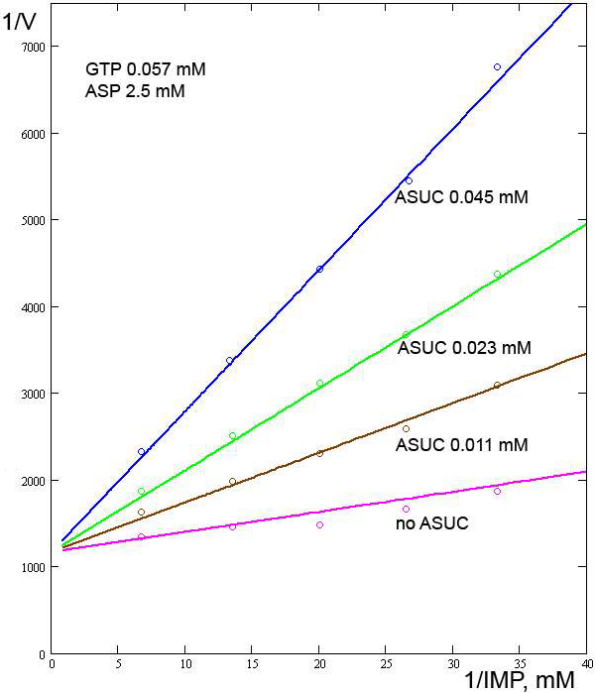
**Relationships between the reaction rate and the IMP concentration in the presence/absence of ASUC**. Experimental data from [7].

**Figure 3:**
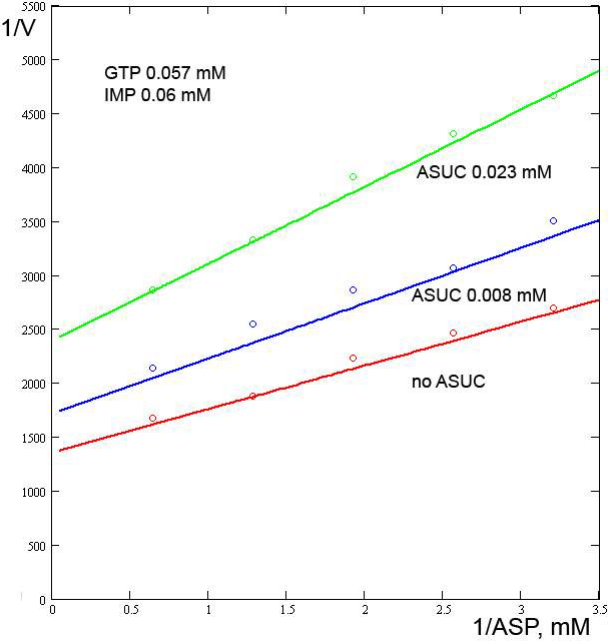
**Relationships between the reaction rate and the ASP concentration in the presence/absence of ASUC**. Experimental data from [7].

Wyngaarden and Greenland [6] investigated the effect of ASUC, another inhibitor, and proposed that it is competitive with both IMP and ASP. Likewise, it was proposed that GMP is a competitive inhibitor of both IMP and GTP. However, our calculations suggest that ASUC appears to be competitive with only IMP, and GMP with only GTP. The effects of ASUC on IMP and ASP and the model output are presented in Figs. 2, 3. Using the model, *Ki*_*ASUC *_is 7.5·10^-3 ^mM.

**Figure 4:**
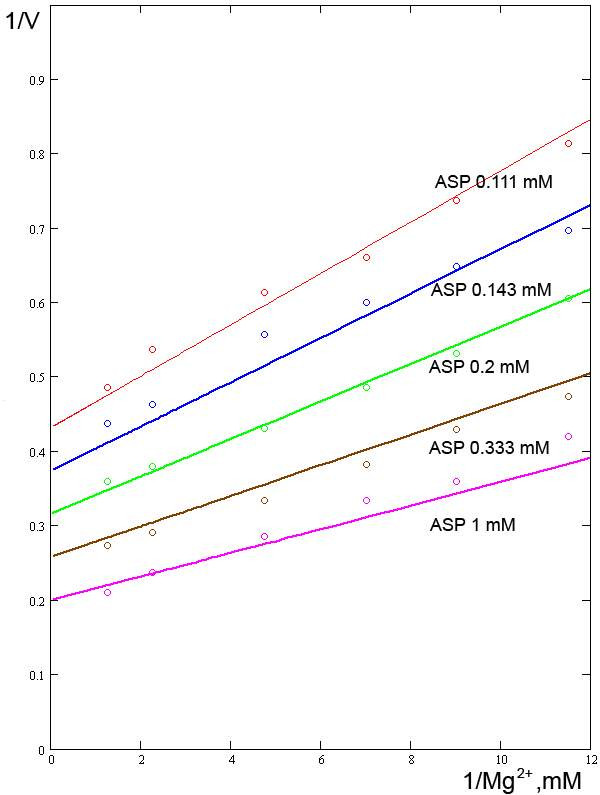
**Relationships between the reaction rate and concentration of Mg2+ ions at varying concentrations of ASP**. Experimental data from [9].

The influence of Mg^2+ ^ions on enzyme activity is included in our model on the basis of the kinetic curves presented in the work of Kang and Fromm [9]. The concentration of Mg^2+ ^is included as a multiplier in the form of a simple rational fraction raised to the first power. Kang and Fromm also included the concentration of Mg^2+ ^as a multiplier raised to the first power; however, they additionally assumed that Mg^2+ ^and ASP may act cooperatively. Our calculations demonstrate that an even simpler model, which does not assume Mg^2+^/ASP synergy, is adequate for describing the influence of magnesium. Although our model does not say that there are two binding sites for Mg^2+ ^ions, nor does it say otherwise [12], for it is not necessary that Hill's number be the same as the number of ligand-binding centers in the enzyme. Using our model, *Km*_*Mg *_is 0.08 mM (Fig. 4).

**Figure 5:**
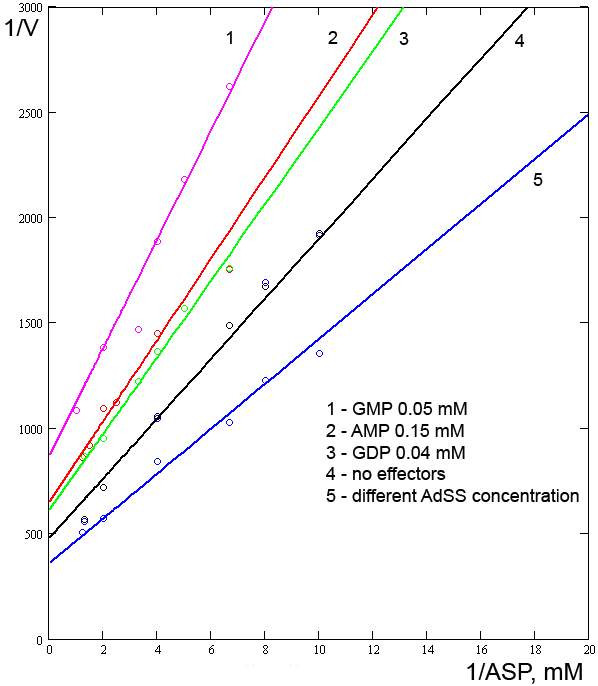
**Relationships between the reaction rate and concentration of ASP under different conditions**. Experimental data from [6].

Also, our model treats AMP as a competitive inhibitor of IMP [6]. From the work of Wyngaarden and Greenland [6], we calculated the constants of the GMP and AMP effects (Fig. 5, lines 1 and 2): *Ki*_*GMP *_= 0.024 mM, *Ki*_*AMP *_= 0.01 mM. However, these estimates may suffer from a lack of consistency within the experimental data.

In control experiments, we used data from Wyngaarden and Greenland [6]. The comparison of the model output and experimental data [6] is presented in Fig. 5, lines 4 and 5. For comparison, the values of the constants obtained here and elsewhere are presented in Table 1.

**Table 1:** Kinetic parameters of Escherichia coli adenylosuccinate synthetase. Our model output, and data provided from the literature.

**Parameter**	**Model output**	**Literature data**	**Reference**
*Km*_*GTP*_	0.023 mM	0.01 mM	7
		0.048 mM	6
		0.0221 mM	10
		0.0262 ± 0.0023 mM	12
*Km*_*IMP*_	0.02 mM	0.02 mM	7
		0.054 mM	6
		0.0243 mM	10
		0.0278 ± 0.0013 mM	12
*Km*_*ASP*_	0.3 mM	0.35 mM	7
		0.225 ± 0.016 mM	9
		0.27 mM	6
		0.191 mM	10
		0.23 ± 0.04 mM	12
*Ki*_SUCC_*	8 mM	-	-
*Ki*_*GDP*_	0.008 mM	0.022 mM	6
*Ki*_*ASUC*_	0.0075 mM	0.27 mM (for IMP)**	6
		0.47 mM (for ASP)	6
*Km*_*Mg*_	0.08 mM	0.114 ± 0.026 mM	9
*Ki*_*GMP*_	0.024 mM	0.092 mM (for GTP)	6
		0.074 mM (for IMP)	6
*Ki*_*AMP*_	0.01 mM	0.095 mM	6

* The effect of SUCC was examined by Rudolph and Fromm [7]. However, their formula is too complicated (it describes the effect of one inhibitor using four different constants) and the inhibition constants were not evaluated.

** In 1963 Wyngaarden and Greenland [6] proposed that ASUC is a competitive inhibitor of both IMP and ASP, and GMP is a competitive inhibitor of both IMP and GTP.

## Discussion

Models to describe the reaction catalyzed by adenylosuccinate synthetase have been proposed from time to time. In 1969, Rudolph and Fromm investigated the mechanism of this reaction and proposed an equation that related the reaction rate to the respective concentrations of the substrates and one inhibitor [7]. However, the formula included four inhibition constants and therefore was too complicated to allow those constants to be evaluated. As a result, only apparent inhibition constants were calculated.

In 1963, Wyngaarden and Greenland [6] proposed that ASUC is a competitive inhibitor of both IMP and ASP. Likewise, it was proposed that GMP is a competitive inhibitor of both IMP and GTP. However, our model, using the same pool of experimental data, demonstrates that the assumption that ASUC is competitive with only IMP, and GMP with only GTP, is sufficient for describing the enzyme reaction adequately.

In 1979, Stayton and Fromm [8] proposed a model that relates the reaction rate to the effect of the inhibitor ppGpp. This model describes the effect of the inhibitor as the above model does, so only the apparent inhibition constants were calculated. In addition, the calculated values of these apparent inhibition constants depend on the substrate concentrations.

In 1995, Kang and Fromm [9] proposed a model that relates the reaction rate to the concentrations of ASP and Mg^2+ ^ions. However, no other substrates or inhibitors were included. Thus, despite all the interest in the reaction catalyzed by AdSS, no single model that includes the concentrations of all substrates and the effects of more than one effector has been proposed. We propose a model that describes the reaction catalyzed by adenylosuccinate synthetase and includes the concentrations of three substrates (GTP, IMP, and ASP), the effects of five inhibitors (GMP, GDP, AMP, ASUC, and SUCC), and the influence of Mg^2+ ^ions. Our model is consistent with a fully random mechanism, which is very similar to that proposed by Rudolph and Fromm [7]. However, given the available biochemical data on the reaction mechanism, at least two alternative hypotheses could be put forward. We checked whether these hypotheses were consistent with the kinetic data.

One of these hypotheses states that magnesium binds to aspartate to form an ASP·Mg^2+ ^complex, which in turn binds to the enzyme. This hypothesis leads to a modification of our model such that it describes the effects of ASP, Mg, and SUCC. To make this modified model consistent with experimental data at a fixed concentration of Mg^2+^, all the parameters, except *Km*_*Mg*_, were assigned the same values as in the original model. The best consistency of the modified model and the experimental data was attained at *Km*_*Mg *_= 0.01 mM. Overall, this modification is much less consistent with the experimental data than the original model (calculations not shown).

The second hypothesis states that ASP binds to the enzyme to form an AdSS·ASP complex, after which magnesium binds to aspartate in that complex. To describe this hypothesis, another modification of the original model is required such that it describes the effects of ASP, Mg, and SUCC. Here the best consistency is attained at *Km*_*Mg *_= 0.13 mM. Overall, this modification too fails to show reasonable consistency with the experimental data (calculations not shown).

Under both modified models (one assuming pre-formation of the ASP·Mg^2+ ^complex, the other assuming pre-formation of the AdSS·ASP complex), magnesium competes against SUCC, while under the original model it does not compete against anything. Looking at the output from the original and modified models, it is easy to see that the original is by far the most consistent. Admittedly, it seems in some respects inconsistent with the available biochemical data: for example, it leaves out the presence of two binding sites for Mg^2+^. Therefore, this model may not be claimed as an absolutely accurate description of the real molecular events unfolding in the reaction being discussed. Looking at the model structure, it appears as though magnesium ions might act on the enzyme via their own independent centers. This hypothetical mechanism therefore deserves to be called an "apparent molecular mechanism" by analogy with apparent dissociation constants, apparent inhibition constants, etc.

As we were working on our model, an inconsistency between the model output and the experimental data on SUCC was revealed (Fig. 1). Since no enzyme concentrations were specified in the literature sources to which we referred, we kept to a fixed arbitrary concentration of the enzyme in all the numerical experiments. However, when we proceeded to SUCC, we found that we had to modify this concentration in order to keep the model consistent with experimental data. We introduced a multiplier equal to 0.74 as a correction factor, which implies a reduction in the concentration of the enzyme (calculations not shown).

The constant *Km*_*Mg *_was verified against data published by Kang and Fromm [9] (Fig. 4). However, to accomplish this, we had to reduce *Km*_*ASP *_two-fold to 0.17 mM and introduce a correction factor (a multiplier equal to 5000), which in effect increases the amount of enzyme. The other model parameters were absolutely consistent with those experimental data.

Some data from Wyngaarden and Greenland [6] were used for control experiments (Fig. 5, lines 4 and 5). On comparing our model output and the control data, an apparent inconsistency was revealed. It is possible that the authors used different amounts of the enzyme in different experiments. This assumption was supported by introduction of a coefficient such as those used for the curves shown in Fig. 1. Admittedly, the discrepancies revealed during the fitting of the parameters could in part be explained by the different temperatures at which the different authors conducted their experiments: Rudolph and Fromm [7] at 28°C, Wyngaarden and Greenland [6] at 25°C, and Kang and Fromm [9] at 22°C. However, in the present work we did not look at temperature as a factor.

## Conclusion

The proposed model for the reaction catalyzed by the enzyme AdSS includes relationships between the reaction rate, the concentrations of three substrates (GTP, IMP and ASP), the effects of five inhibitors (GMP, GDP, AMP, ASUC and SUCC) and Mg^2+ ^ions. Our model is consistent with a fully random mechanism. The model structure implies that each of the inhibitors included in it is competitive to only one of the substrates. The model's parameters were fitted to experimental data from published literature. A methodological problem arising from the lack of concordance among the data in different publications was dealt with by introducing correction coefficients; this simply implies that the concentrations of AdSS in those source works were different. The adequacy of the model was ensured by comparing the theoretical calculations and the experimental data from the literature sources that were not used while the fitting procedure was under way. The values obtained for the parameters are shown in Table 1.

## Methods

The model to describe the reaction catalyzed by adenylosuccinate synthetase was developed using a random biochemical system as follows:

(a)E(0,y,z,w)+X⇔KXE(X,y,z,w)(b)E(x,0,z,w)+Y⇔KYE(x,Y,z,w)(c)E(x,y,0,w)+Z⇔KZE(x,y,Z,w)(d)E(x,y,z,0)+W⇔KWE(x,y,z,W).
 MathType@MTEF@5@5@+=feaafiart1ev1aaatCvAUfKttLearuWrP9MDH5MBPbIqV92AaeXatLxBI9gBaebbnrfifHhDYfgasaacH8akY=wiFfYdH8Gipec8Eeeu0xXdbba9frFj0=OqFfea0dXdd9vqai=hGuQ8kuc9pgc9s8qqaq=dirpe0xb9q8qiLsFr0=vr0=vr0dc8meaabaqaciaacaGaaeqabaqabeGadaaakeaafaqabeabcaaaaeaadaqadaqaaiabbggaHbGaayjkaiaawMcaaaqaaiabdweafnaabmaabaGaeGimaaJaeiilaWIaemyEaKNaeiilaWIaemOEaONaeiilaWIaem4DaChacaGLOaGaayzkaaGaey4kaSIaemiwaG1aaCbiaeaacqGHuhY2aSqabeaacqWGlbWsdaWgaaadbaGaemiwaGfabeaaaaGccqWGfbqrdaqadaqaaiabdIfayjabcYcaSiabdMha5jabcYcaSiabdQha6jabcYcaSiabdEha3bGaayjkaiaawMcaaaqaamaabmaabaGaeeOyaigacaGLOaGaayzkaaaabaGaemyrau0aaeWaaeaacqWG4baEcqGGSaalcqaIWaamcqGGSaalcqWG6bGEcqGGSaalcqWG3bWDaiaawIcacaGLPaaacqGHRaWkcqWGzbqwdaWfGaqaaiabgsDiBdWcbeqaaiabdUealnaaBaaameaacqWGzbqwaeqaaaaakiabdweafnaabmaabaGaemiEaGNaeiilaWIaemywaKLaeiilaWIaemOEaONaeiilaWIaem4DaChacaGLOaGaayzkaaaabaWaaeWaaeaacqqGJbWyaiaawIcacaGLPaaaaeaacqWGfbqrdaqadaqaaiabdIha4jabcYcaSiabdMha5jabcYcaSiabicdaWiabcYcaSiabdEha3bGaayjkaiaawMcaaiabgUcaRiabdQfaAnaaxacabaGaeyi1HSnaleqabaGaem4saS0aaSbaaWqaaiabdQfaAbqabaaaaOGaemyrau0aaeWaaeaacqWG4baEcqGGSaalcqWG5bqEcqGGSaalcqWGAbGwcqGGSaalcqWG3bWDaiaawIcacaGLPaaaaeaadaqadaqaaiabbsgaKbGaayjkaiaawMcaaaqaaiabdweafnaabmaabaGaemiEaGNaeiilaWIaemyEaKNaeiilaWIaemOEaONaeiilaWIaeGimaadacaGLOaGaayzkaaGaey4kaSIaem4vaC1aaCbiaeaacqGHuhY2aSqabeaacqWGlbWsdaWgaaadbaGaem4vaCfabeaaaaGccqWGfbqrdaqadaqaaiabdIha4jabcYcaSiabdMha5jabcYcaSiabdQha6jabcYcaSiabdEfaxbGaayjkaiaawMcaaiabc6caUaaaaaa@AC2E@

Equation (a) describes the interaction of GTP or GDP or GMP with its respective active center. It is assumed that GTP, GDP and GMP compete against one another for binding to the same center, which is why *X *stands for GTP or GDP or GMP. It is assumed that the population of the other enzyme centers has no effect on reactions (a). Equation (b) describes the interaction of IMP or AMP or ASUC with a different active center. *Y *stands for IMP or AMP or ASUC. Equation (c) describes the interaction of ASP and SUCC with a third active center, *Z *standing for either ASP or SUCC. Finally, Equation (d) describes the interaction of magnesium ions with the enzyme; here *W *stands for Mg. *E(x, y, z, w) *describes the state of the enzyme: *x *is assigned 0, GTP, GDP or GMP; y is assigned 0, IMP, AMP or ASUC; z is assigned 0, ASP, and SUCC; *w *is assigned either 0 or Mg. If a variable in *E(x, y, z, w) *takes on a zero value, it means that the corresponding enzyme center is not bound to the ligand.

Owing to the assumption that the system is random, the variables *X, x, Y, y, Z, z, W, w *in Equations (a)-(d) are always independent. Therefore, Equations (a) and (b) each include 72 reactions and Equation (c) includes 64 reactions. Equation (d) is a concise description of 48 reactions. Overall, the system (a)-(d) includes 256 biochemical reactions that describe transitions among 96 states of the enzyme. Writing out the corresponding system of 96 differential equations and assuming equilibrium results in a non-linear algebraic system. Supposing that variation in the concentrations of GTP, GDP, GMP, IMP, AMP, ASUC, ASP, SUCC and Mg^2+ ^can be neglected, we arrive at the corresponding system of linear equations defining a 96-dimensional vector of the unknown states of the enzyme. Solving this system and collecting similar terms and assuming that *Km*_*GTP *_≡ *K*_*GTP*_, *Ki*_*GDP *_≡ *K*_*GDP*_, *Ki*_*GMP *_≡ *K*_*GMP*_, *Km*_*IMP *_≡ *K*_*IMP*_, *Ki*_*AMP *_≡ *K*_*AMP*_, *Ki*_*ASUC *_≡ *K*_*ASUC*_, *Km*_*ASP *_≡ *K*_*ASP*_, *Ki*_*SUCC *_≡ *K*_*SUCC*_, *Km*_*Mg *_≡ *K*_*Mg*_, we obtain the proposed model.

## Abbreviations

AdSS, adenylosuccinate synthetase; AMP, adenosine 5'-monophosphate; ASP, aspartate; ASUC, adenylosuccinate; CMP, cytidine-5'-monophosphate; dAMP, deoxyadenylic acid; dGMP, deoxyguanylic acid; GDP, guanosine 5'-diphosphate; GMP, guanosine 5'-phosphate; GTP, guanosine 5'-triphosphate; IMP, inosine 5'-monophosphate; PI, phosphate; ppGpp, guanosine 5'-diphosphate-3'-diphosphate; SUCC, succinate; UMP, uridine 5'-monophosphate.

## Competing interests

The author(s) declare that they have no competing interests.

## Authors' contributions

OEA was responsible for developing of the model, and writing of the manuscript.

LVA was responsible for developing of the modelling method and critical review of the manuscript.
